# Direct Speaker Gaze Promotes Trust in Truth-Ambiguous Statements

**DOI:** 10.1371/journal.pone.0162291

**Published:** 2016-09-19

**Authors:** Helene Kreysa, Luise Kessler, Stefan R. Schweinberger

**Affiliations:** 1 Department of Psychology, General Psychology and Cognitive Neuroscience, Friedrich Schiller University Jena, Jena, Germany; 2 DFG Research Unit Person Perception, Friedrich Schiller University Jena, Jena, Germany; University of Leicester, UNITED KINGDOM

## Abstract

A speaker’s gaze behaviour can provide perceivers with a multitude of cues which are relevant for communication, thus constituting an important non-verbal interaction channel. The present study investigated whether direct eye gaze of a speaker affects the likelihood of listeners believing truth-ambiguous statements. Participants were presented with videos in which a speaker produced such statements with either direct or averted gaze. The statements were selected through a rating study to ensure that participants were unlikely to know a-priori whether they were true or not (e.g., “sniffer dogs cannot smell the difference between identical twins”). Participants indicated in a forced-choice task whether or not they believed each statement. We found that participants were more likely to believe statements by a speaker looking at them directly, compared to a speaker with averted gaze. Moreover, when participants disagreed with a statement, they were slower to do so when the statement was uttered with direct (compared to averted) gaze, suggesting that the process of rejecting a statement as untrue may be inhibited when that statement is accompanied by direct gaze.

## Introduction

The function of our eyes is not just limited to seeing and perceiving the world around us. Through the eyes, humans send a multitude of signals to the people in their environment, providing information on current focus of attention, behavioural intentions, as well as emotional and mental states (for reviews, see [1–4]). From earliest infancy onwards, humans are able to discriminate between direct and averted gaze in others and, in fact, show enhanced processing when someone is looking at them directly ([5], see also [6], for further evidence of the facilitatory effect of direct gaze in older children). This importance of being looked at and of resulting instances of mutual gaze is of course especially relevant in the context of direct face-to-face social interaction, where evaluations of a communicative partner’s personality can be affected by their gaze behaviour. For example, static images of faces showing direct gaze, or faces shifting gaze towards the perceiver, have been shown to appear more attractive [7,8], more likeable [9], and more trustworthy [10–12]. They are also more likely to be remembered [13,14]. Interestingly, perceptions of attractiveness and eye contact also appear to interact with each other [15,16].

However, direct gaze is not necessarily always a pleasant thing: Being attended to closely leads to heightened arousal [17,18], presumably facilitating the preparation of an appropriate response. It is easy to imagine situations in which being scrutinised by a stranger, a person with authority, an entire crowd, or a sabre-toothed tiger feels distinctly uncomfortable [19]. Not surprisingly, direct gaze therefore plays an important role in threat gestures in many primate societies and among other species [3,20–22]. This hostile aspect of direct gaze stems from its connection with dominance (or asserted dominance) of the individual producing the gaze behaviour. In line with the idea that shy or socially anxious people are more likely to avoid gaze contact [23], faces showing direct gaze were generally rated higher on dominance than faces with averted gaze, and this was particularly so if the shape of the face in question had been masculinised to enhance dominant characteristics [24].

Thus, whether direct gaze is experienced as positive or negative depends among other things on the personal relevance of the situation [9] and the mood of both the producer and the perceiver of the gaze [11,12]. One area where this ambiguity of direct gaze is particularly evident is the assessment of an individual’s trustworthiness based on their gaze behaviour. It is often believed to be well-established that liars are likely to avert their gaze and avoid gaze contact [25]. Actually, this is notoriously difficult to investigate in a controlled setting [26]. Moreover, and at variance with this belief, a liar who is aware of this commonly held assumption should make a particular effort to establish eye contact with conversational partners. In fact, in a recent study in which airport passengers were asked either to tell the truth or to lie about their forthcoming trip, liars have indeed been reported to display more eye contact than truth tellers [27]. Nevertheless, a large-scale cross-cultural analysis of beliefs about deceptive behaviour found gaze aversion to be the factor most frequently named as an index of lying [28]. Relatedly, viewers tasked with detecting whether a speaker was lying or telling the truth have been shown to spend a large proportion of total viewing time fixating the speaker’s eye region, yet this did not correlate with whether they correctly detected deception [29]. In sum, there seems to be considerable discrepancy between stereotypes about liars’ gaze behaviour on the one hand, and liars’ actual gaze behaviour on the other hand.

In the present research, rather than focussing on intentional lying, we were more interested in influences of gaze behaviour on the persuasiveness of factual statements with unclear truth value. Of relevance, Swerts and Krahmer [30] reported that speakers who lacked confidence about the correctness of their statements were more likely to avert their gaze than those who knew they were correct (see also [31]). With regard to the perspective of the listener, Chen, Minson, Schöne and Heinrichs [32] studied listeners’ willingness to meet speakers’ gaze depending on whether they agreed with the speaker’s opinion. Assuming that the speaker looks at the listener while making a controversial statement, it is the listener whose gaze behaviour–either meeting or avoiding the speaker’s gaze–decides whether eye contact and mutual gaze are established. Chen and colleagues reasoned that precisely because of the association of direct gaze with persuasion, dominance and intimidation, instances of eye contact might actually make listeners less likely to amend their own prior opinion and agree with the speaker. In two studies, listeners were eyetracked while they watched videos of speakers making persuasive arguments for personally-held beliefs on controversial topics, such as nuclear energy or university tuition fees. Listeners more frequently looked at the speaker’s eyes when they agreed with the speaker’s opinion; however, if they disagreed with the speaker, longer fixation of the speaker’s eye region made them less likely to change their own opinion towards that of the speaker.

The present study focused on how speaker gaze affects evaluations of factual statements for which listeners were unlikely to know a-priori whether they are true or not. Considering the finding that a speaker who is unsure about the truth-value of a statement is likely to avert gaze, we investigated whether direct gaze affected the believability of the content uttered by the speaker. In particular, we expected that direct eye gaze, as compared to averted eye gaze, would make the same ambiguous statements more credible and therefore lead to a higher number of “yes”-belief responses. We also analysed response times as a continuous measure of temporal facilitation or inhibition for making these judgments depending on the speaker’s eye gaze.

## Methods and Materials

### Participants

**Fig 1:**
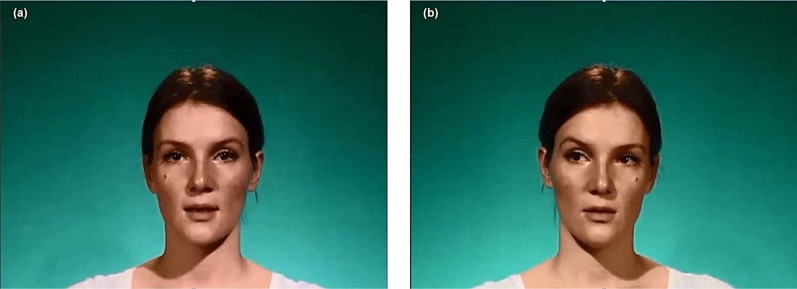
Example screenshots from the videos. (a) shows the speaker speaking with direct gaze, (b) is from a mirrored version with left-averted gaze. The individual depicted here provided written consent regarding the publication of her photograph in this form.

**Fig 2:**
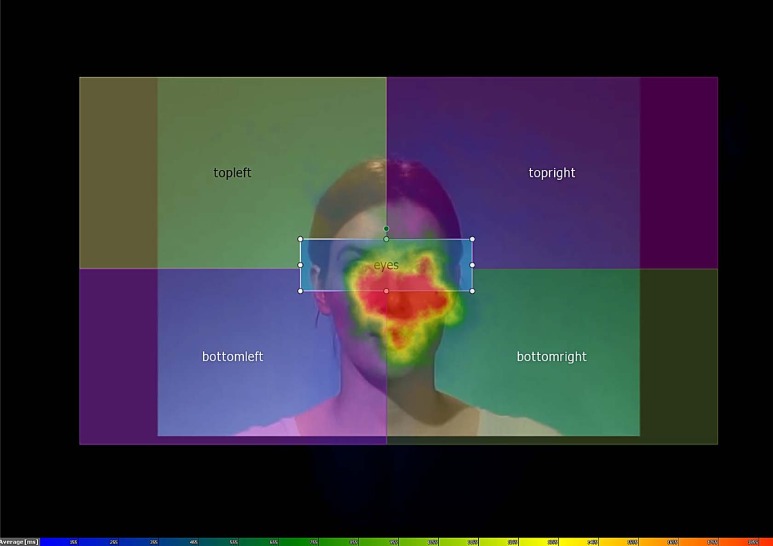
Heatmap depiction of fixation patterns for 5000 ms from the start of each video, across all participants and conditions. Warm colours indicate long total fixation times, cool colours short fixation times. Fig 2 also depicts the five regions of interest used for analysis, although the speaker’s eye region is largely hidden by the large blob of fixations. Please note that due to small shifts in the position of the speaker’s head during speaking, the regions of interest are approximations of the true position. The individual depicted here provided written consent regarding the publication of her photograph in this form.

Data from 35 students of the Friedrich Schiller University of Jena (4 male, mean age 22, range 17–37) were used in the analyses. Data from one additional participant were excluded from all analyses, because her response pattern suggested insufficient task compliance (consistent “yes”- and “no”-responses to all direct-gaze and averted-gaze statements, respectively). All participants had normal or corrected-to-normal vision and took part in exchange for course credit. Participants were recruited through an email announcing an experiment on media competence. All participants provided written informed consent. For the only participant who was under-age, additional signed consent of legal caretakers was provided via enrolment in the psychology curriculum, of which partaking in experiments is a mandatory part. The study was carried out in accordance with the Declaration of Helsinki and was approved by the ethics commission of the Faculty of Social and Behavioural Sciences at the Friedrich Schiller University of Jena (approval no. FSV 15/16). The individual depicted in Figs 1 and 2 gave written informed consent using the PLOS consent form to publish these images.

### Materials

#### Statements

Our stimuli consisted of 36 statements which were deliberately selected so that our student sample was unlikely to know a-priori whether they were true or not. To give one example, consider the statement “sniffer dogs cannot tell the difference between identical twins”. Although recent research suggests that in fact they can [33], the actual truth of statements was irrelevant for the present purpose. Rather, we took measures to ensure that any such knowledge was generally inaccessible to our participants. To obtain experimental statements, we initially collected a set of 50“weird facts”, mostly from the trivia page of a local magazine (Stadtmagazin Jena 07). Because we wanted to include only statements for which our participants would be unsure of the truth-value (irrespective of the actual correctness of their beliefs), we ran a pilot rating study using two paper-and-pencil lists, each of which contained 25 of these “weird facts” and 25 false statements corresponding to the 25 weird facts on the other list. The false statements were created by changing a word or a number in each weird fact statement. Pilot participants (*N* = 42), none of whom participated in the main study, each received one of these two lists and were told to read the statements and indicate as quickly as possible whether or not they believed them on a four-point scale (0 =“definitely not true”, 1 =“probably not true”, 2 = “probably true”, 3 =“definitely true”). We then computed mean ratings for each “weird fact”. This allowed us to select only the most truth-ambiguous statements for the main study, with mean ratings between 1 and 2 and less than seven individual ratings of 0 or 3 (for the resulting items: *M* = 1.58, *SD* = 0.23). Please see the Appendix for a list of all statements used in the study.

#### Videos

Subsequently, we video-recorded a female speaker who uttered each statement in two conditions: direct and averted eye gaze. For the direct gaze recording, the speaker looked at a fixed point just above the camera, creating the impression that she was fixating the participant directly. For the averted gaze condition, she briefly looked at the camera and then shifted her gaze to a fixed point about 10 degrees to the right side of the camera before starting to speak. Head movements were kept minimal. The speaker repeated the statements after hearing them read out to her (to avoid any eye movements that might have been due to reading statements). She endeavoured to speak with the same, relatively neutral stress pattern in both conditions and to maintain a comparable timing and rhythm. We recorded each statement three times in each condition in order to select the best versions. The resulting 72 videos were cut and mirrored to create a left-averted gaze condition (the lighting arrangement in our recording set-up would have made it difficult to record left-averted gaze in the same way as right-averted gaze). On average, the videos lasted *M* = 5.68 seconds (*SD* = 1.47). Example screenshots are displayed in Fig 1.

### Design and Procedure

The independent variable was the manipulation of gaze direction, with a single speaker uttering all statements in all three gaze conditions (direct gaze, right-averted, left-averted). The experiment used a within-subjects design: Each of the 36 statements was played once to every participant, in such a way that each participant saw one third (i.e. 12) of the statements in the direct-gaze video condition, one third in the right-averted and one third in the left-averted gaze condition. Statements were presented in random order. Three experimental lists ensured that across participants, every statement was presented equally frequently in all three conditions. In other words, every statement occurred only once per list, and every list contained equal numbers of statements in each condition.

To ensure that participants actually looked at the speaker’s face, the experiment was run using an SMI iViewX Hi-Speed eye-tracker. Participants arrived at the laboratory individually and were greeted by one of three female experimenters (one experimenter was not a native speaker of German, so all three used closely scripted instruction sheets). After participants had signed the consent form, we determined their eye-dominance using the Porta-Rosenbach test ([34] left-dominant 12, right-dominant 23). Next, they were seated comfortably in front of the eye-tracker and were calibrated using a 9-point calibration and 4-point validation routine in SMI ExperimentCenter (v. 3.4.165). Calibration was defined as successful if the maximum error was smaller than 1°; eye movements were recorded at 500 Hz.

The actual experiment was programmed in PST E-Prime (v. 2.0.8.22) and began with a demographic registration of age, gender, handedness and eye-dominance. This was followed by on-screen instructions and two practice trials to ensure that participants had understood the procedure. Participants were then presented with the 36 videos of their respective list, in randomised order. Each video was preceded by a central fixation cross for 500 ms and immediately followed by the question *Do you believe this*? (“Glaubst du das?”). This question remained in the centre of the screen until participants pressed one of two response keys (either *yes* or *no)* using their index fingers, at which point the next fixation cross appeared. For half the participants, the agree button was on the right side, for the other half on the left.

Following the main experiment, participants were asked to rate the speaker on a 6-point scale with regard to six person attributes *(The speaker is… [adjective]*: *attractive*, *believable*, *competent*, *intelligent*, *likeable*, *trustworthy;* the original German adjectives were “attraktiv”, “glaubwürdig”, “kompetent”, “intelligent”, “sympathisch”, “vertrauenswürdig”). These were assessed in randomized order.

Thus, the main dependent variables were participants’ belief in the truth of each statement (measured through the number of belief responses), their response times (measured from the onset of the question screen) as an indicator of how sure they were about their response, and the subsequent ratings of the speaker. The experiment concluded with a post-experimental paper-and-pencil questionnaire containing all 36 statements, on which participants were asked to mark any that they had prior knowledge about. The post-experimental questionnaire also included three debriefing questions: whether they had noticed any regularities, patterns or unusual events during the experiment, what they believed was the aim of the study, and whether they had used any particular strategies to prepare their responses. The entire session lasted about 20 minutes.

### Control experiment

We also ran a control experiment to address the possibility that the statements in the averted and direct gaze conditions differed in some way other than the speaker’s gaze (for example, something in the speaker’s voice could have made participants believe statements more in one condition than in the other). We tested 37 additional students (6 male; mean age 23, range 18–45) who performed the exact same task on the same materials, but with auditory presentation only. Instead of the video frames, the screen showed a constant fixation cross on a grey background.

## Results

Please note that where appropriate, we performed epsilon corrections for heterogeneity of covariances using the Huynh-Feldt procedure throughout [35].

### Control experiment

In the control experiment with auditory presentation only, participants believed the statements in 55% of trials. Importantly, approximately equal numbers of “yes”-responses were produced for the statements that came from videos with direct gaze (*M* = 10.05 out of 18, *SD* = 2.32) than from those with averted gaze (*M* = 9.62, *SD* = 2.36). A one-way repeated-measures ANOVA on the mean number of “yes”-responses per gaze condition (direct vs. averted) showed no significant effect (*F*(1,36) = 0.26, *p* = .259, *η*² = .009). Accordingly, for auditory-only presentation, the speaker’s gaze direction in the original video had no effect on listener belief. We are therefore confident that any effect of speaker gaze on belief responses in the main experiment does not reflect subtle differences in vocal patterns during the production of these statements.

### Belief responses

In the main experiment, participants indicated that they believed the speaker’s statements in roughly half the trials (48.3%; range 13–23 “yes”-responses out of 36). Crucially, the speaker’s gaze behaviour clearly affected whether listeners believed her statements: As Table 1(A) reveals, more “yes”-responses were made following videos with direct gaze than averted gaze. A comparison of the mean number of “yes”-responses per condition in a one-way repeated-measures ANOVA reveals a main effect of gaze direction (*F*(2, 68) = 5.45, *p* = .011, *ε* = 0.81, *η*² = .103): On average, participants responded “yes” more often for direct gaze (*M* = 6.71 out of 12, *SD* = 2.07) than for averted gaze trials (left-averted: *M* = 5.46, *SD* = 1.92; right-averted: *M* = 5.20, *SD* = 1.95). Please note that although we did not predict differences in “yes”-responses between left-averted and right-averted trials, they were analysed separately to ensure equal numbers of trials in all conditions. In fact, Bonferroni-corrected post-hoc t-tests revealed a marginally significant difference between left-averted and direct gaze trials (*p* = .061) and a significant difference between right-averted and direct gaze trials (*p* = .040), but no difference between the two averted gaze conditions (*p* > .9).

**Table 1:** Listeners' acceptance of the speaker's statements by gaze condition for (a) the total sample of *N* = 35 participants, as well as for (b) the subset of participants who made no mention of gaze direction in the debrief questionnaire (*N* = 11), and (c) the subset of those who did (*N* = 24).

Gaze condition	(a) Frequency of “yes”-responses (out of 420 responses per condition)	(b) Subgroup of “naïve” participants: Frequency of “yes”-responses (out of 132 responses per condition)	(c) Subgroup of “gaze-mentioning” participants: Frequency of “yes”-responses (out of 288 responses per condition)
**averted left**	**191 (45.5%)**	53 (40.2%)	138 (47.9%)
**averted right**	**182 (43.3%)**	57 (43.2%)	125 (43.4%)
**direct**	**235 (56.0%)**	65 (49.2%)	170 (59.0%)
**Total**	**608 (48.3%)**	175 (44.1%)	433 (50.1%)

To address whether the key finding of more “yes”-responses following direct than averted gaze might have been influenced by participants suspecting that the study was about influences of gaze direction, we split the sample into two subgroups depending on whether they did (*N* = 24) or did not (*N* = 11) mention gaze direction in the debrief questionnaire. An ANOVA with an additional between-subjects factor of subgroup confirmed a main effect of gaze direction (*F*(2, 66) = 5.39, *p* = .007, *ε* = 0.81, *η*² = .107), and a tendency for more “yes”-responses overall in the gaze-mentioning subgroup (*F*(1, 33) = 3.73, *p* = .06, *η*² = .029). Critically, there was no hint of an interaction between gaze direction and subgroup (*F* < 1), indicating a similar effect of direct gaze in both subgroups (see Table 1).

For a minority of statements (*M* = 3.6 per participant out of 36 trials), participants indicated on the post-experimental questionnaire that they had prior knowledge about the truth-value. We therefore repeated these analyses while excluding such trials. As the pattern of results from this analysis was equivalent to the findings when including all trials, we decided to report the results based on the entire data set.

### Response times

Response time (RT) data from one participant could not be used due to a missing cell (no “yes”-responses after averted left videos). Due to the typical right skew of RT distributions, all values were log-transformed for further analyses; however, descriptive statistics will be provided in milliseconds for ease of interpretation (see Tables 2 and 3). Outliers beyond 3 *SD* from mean log RT were excluded (approx. 1% of trials). A 2×3 repeated-measures ANOVA revealed a main effect of gaze direction (*F*(2, 66) = 7.48, *p* = .001, *ε* = 1.00, *η*² = 0.015), but no main effect of response type (*F*(1, 33) = 1.77, *p* = .192, *η*² = 0.003). Importantly, there was a significant interaction of gaze direction and response type (*F(*2, 66) = 4.12, *p* = .021, *ε* = 0.94, *η*² = 0.011). To explore this interaction further, we split the data by response type. Bonferroni-corrected pairwise t-tests revealed that participants were slower to respond “no” following a statement produced with direct gaze than in the right-averted condition (*p* < .001), and marginally slower with direct gaze than in the left-averted condition (*p* = .09). None of the differences between conditions reached significance for “yes”-responses.

**Table 2:** Mean response times in ms by gaze condition and response (*SEM* in parentheses; *N* = 35).

Gaze direction	Mean RT in ms by response type (*SEM)*		Total
	“yes”	“no”	
averted left	819 (*77*.*3*)	793 (*69*.*4*)	806 (*51*.*5*)
averted right	776 (*68*.*7*)	753 (*79*.*6*)	766 (*52*.*2*)
direct	780 (*68*.*0*)	946 (*100*.*4*)	863 (*61*.*0*)
Total	792 (*40*.*8*)	831 (*48*.*8*)	811 (*31*.*8*)

**Table 3:** Audio-only control experiment: Mean response times in ms by (original) gaze condition and response type(*SEM* in parentheses; *N* = 37).

Original gaze direction	Mean RT in ms by response type (*SEM)*		Total
	“yes”	“no”	
averted	679 (*33*.*2*)	694 (*32*.*2*)	686 (*23*.*2*)
direct	731 (*33*.*8*)	760 (*36*.*9*)	743 (*24*.*9*)
Total	705 (*23*.*7*)	726 (*24*.*4*)	715 (*17*.*0*)

Finally, when splitting participants into subgroups defined as above, the main effect of gaze direction was confirmed, with slower responses to direct than averted gaze (*F*(2, 64) = 3.87, *p* = .025, *η*² = .008). Independent of gaze direction, there was an interaction of subgroup and response type (*F*(1, 32) = 8.90, *p* = .005, *η*² = .012): While “yes”-responses were faster than “no”-responses in the gaze-mentioning subgroup (*M*_“yes”_ = 756 ms, *SD* = 370; vs. *M*_“no”_ = 865 ms, *SD* = 510), the reverse pattern was found in naïve participants (*M*_“yes”_ = 886 ms, *SD* = 403; vs. *M*_“no”_ = 779 ms, *SD* = 333). Importantly however, there was neither a main effect of subgroup, nor any higher-order interaction between subgroup and gaze direction (all *F*-values < 1).

In the audio-only control experiment, a 2×2 repeated-measures ANOVA revealed a significant main effect of original gaze direction: Responses following trials from videos with direct gaze were slower than to those that had originally been shown with averted gaze (*F*(1,36) = 7.14, *p* = .011, *η*² = 0.016; see Table 3 for descriptive statistics). There was also a marginal effect of response type (*F*(1, 36) = 3.17, *p* = .084, *η*² = 0.006), with “yes”-responses being made slightly faster than “no” responses. Importantly, there was no interaction between original gaze direction and response type (*F* < 1). Thus, although the results of the control experiment raise the possibility that subtle acoustic differences between statements produced with direct vs. averted gaze may have influenced RTs overall, any such influence cannot explain the interaction of gaze direction and response type in the main experiment.

### Fixation patterns

We also performed a coarse analysis of participants’ fixation patterns to the videos of the speaker. We used SMI BeGaze (v. 3.4.52) to define five static regions of interest (the speaker’s eye region and four equal-sized screen segments, see Fig 2). We compared total fixation time in these regions between gaze conditions and depending on participants’ responses. In total, participants fixated slightly longer in the non-eye regions (*M* = 2800 ms, *SD* = 1395) than in the eye region (*M* = 2184 ms, *SD* = 1321). This pattern was not consistently affected either by the speaker’s gaze behaviour, or by whether participants believed her statement. Fig 2 suggests that fixations in the present experiment were distributed across the entire area of the face including the mouth, whereas other studies using static faces tend to find a hotspot of fixations in the eye region [36]. In our view, this may be attributed to our use of dynamic speaking faces, in which the articulating mouth region may attract fixations.

### Post-experimental ratings

Finally, we analysed whether the tendency to agree with the speaker’s statements correlated with participants’ post-experimental rating of the speaker on six person attributes: Across participants, the speaker was rated relatively highly on a five-point scale for attractiveness (*M* = 4.64, *SD* = 1.02), likeability (*M* = 4.53, *SD* = 1.13), intelligence (*M* = 4.28, *SD* = 0.88), competence (*M* = 3.92, *SD* = 0.97), trustworthiness (*M* = 3.81, *SD* = 1.01), and believability (*M* = 3.64, *SD* = 0.83; attributes in descending order of mean ratings). However, none of these ratings correlated with the number of trials on which participants indicated that they believed what the speaker had said (all *p*s > .29 for Kendall’s *τ*).

## Discussion

The present experiment investigated the effects of direct vs. averted speaker gaze on whether listeners believed a speaker’s truth-ambiguous statements. We found that listeners were substantially more likely to believe a statement made with direct gaze, as compared to averted gaze. Moreover, only in those cases in which participants disagreed with a statement which had been uttered with direct gaze, they showed increased response times, compared to when disagreeing with a statement made with averted gaze. These findings suggest that direct gaze can bias perceivers towards accepting a truth-ambiguous statement as being true. At the same time, when participants judged a statement as being false (perhaps due to partial knowledge or pre-experimental beliefs), direct gaze appeared to interfere with the rejection of that statement.

The present paper established an experimental paradigm that allowed us to demonstrate a novel effect of direct speaker gaze on subjective credibility of truth-ambiguous statements. While the present approach therefore seems promising in order to study gaze effects in relatively naturalistic situations of human interaction, our results extend previously reported effects of direct gaze on impressions about people based on static images. For instance, direct gaze appears to increase trustworthiness (e.g., [11,12]), a notion which is widely held in the non-scientific community. We should like to note that while the present study establishes strong effects of a speaker´s gaze direction on the evaluation of truth-ambiguous statements, the degree to which direct gaze can be a valid and “honest signal” for trustworthiness across situations cannot be addressed by studies of this kind. To some extent, an analogous situation holds for a number of other studies showing positive effects of direct gaze on diverse social evaluations, such as attractiveness ratings [7,8], likeability [9], and memory for a particular face [9,14].

Relatedly, the reasons and mechanisms behind a “trustworthiness-bonus” of direct gaze are largely unclear (see [37] and [1], for reviews on the neural processing of eye contact). Adams and Kleck ([38], but see [39] for qualifications) suggested that, like emotional expressions, gaze direction is a signal of motivation for approach (direct gaze) or avoidance (averted gaze) behaviour. This could account for the context-dependency of gaze in social situations: Direct gaze is particularly salient in approach-related contexts (positive: happy or attractive faces, negative: angry faces), while averted gaze facilitates the identification of fearful and sad faces [1]. In the context of decisions on whether to believe another person, direct vs. averted gaze might thus be interpreted differentially, depending on whether the listener believes the speaker is trying to persuade them (approach motivation; [32]) or is looking away because s/he is also not convinced of the truth value of a statement (avoidance; [30]).

Tentative support for this interpretation comes from natural pedagogy theory [40], according to which direct gaze functions as a highly salient cue of communicative intent. Specifically, direct gaze is assumed to generate a strong expectation in the perceiver that she or he will be taught an item of generalizable knowledge–a mechanism which might well be invoked when listeners are assessing the content of general knowledge statements like the ones used in our study. We deliberately selected statements about which listeners were unlikely to have strong prior opinions, reasoning that a “pure” effect of the speaker’s gaze behaviour should be easier to detect when listeners were not sure of the answer themselves. Imagine for example, that the speaker says “four times four is sixteen” or “four times four is nine”–her gaze would hardly be expected to play any role for deciding on whether either of these statements is true or false. In contrast, if listeners have no prior knowledge and are thus forced to guess the correct answer, as in the present study, they may be inclined to make use of any available cues to help them in their decision.

The finding that participants were faster to reject a statement produced with averted gaze than a statement with direct gaze was not expected and, to the best of our knowledge, has not been described before. A plausible interpretation is that participants are confronted with a form of cue-conflict in this situation: On the one hand, the speaker’s gaze and demeanour is persuasive; on the other hand, given their world knowledge they really find the semantic content of the sentence unlikely. Resolving this conflict appears to require additional analysis and may be more effortful than agreeing with the speaker, in line with models of response time in situations of competing information [41].

One limitation of our study may be the use of one speaker for all sentences and in both gaze conditions. This was a deliberate decision in order to minimise effects of differentially trustworthy faces on whether participants believed the statements [42]. However, we do not know whether our speaker was actually representative of the population norm or may have appeared more (or less) trustworthy a-priori. In fact, because participants experienced her speaking with averted gaze on two-thirds of trials, it is possible that our post-experimental ratings of her trustworthiness may slightly underestimate their pre-experimental first impressions. Additionally, the fact that she shifted gaze on some trials but not on others made the design relatively transparent and may have increased the tendency to respond consistently. According to the post-experimental questionnaire, about two-thirds of the participants guessed that we might have been interested in effects of eye gaze direction. Note however, that a general response tendency contingent on gaze direction cannot explain participants’ increased response times for rejecting direct-gaze statements. If response times in this task would reflect a general response tendency, one would also have expected faster responses for accepting direct gaze statements, which we did not find (see Tables 2 and 3).

Relatedly, although the use of a single speaker ensured that our stimuli were as similar as possible across conditions, the extent to which our results generalise to the general population of potential speakers remains to be studied. An intriguing extension of this research could be to use different gaze-directions contingent upon different speaker identities, since this could reveal whether characteristic gaze behaviours may affect perceived individual trustworthiness. It should also be noted that with only four male participants (vs. 31 females, 11%) participant gender was not balanced in our study. Sex-differences have been found in the past with regard to gaze-following [43,44]. In this context, potential sex-differences for effects of gaze behaviour on perceived trustworthiness may warrant further investigation.

The finding that participants were slightly but consistently slower in responding to left-averted compared with right-averted videos was likely caused by the fact that we had produced the left-averted condition by mirroring the right-averted videos. Apart from gaze, this manipulation also mirror-reversed any other minor facial and image asymmetries and might have incurred small recalibration costs on the perceiver´s part. However, it is important to note that effects of response type were equivalent for left-averted and right-averted videos. We therefore may assume that any such recalibration costs were constant, and unrelated to the present study’s main findings.

In conclusion, the present study has established a powerful effect of direct speaker gaze on judging truth-ambiguous statements in a condition in which speaker identity was kept constant. In future research, it would be interesting to investigate the interplay between gaze information and other social signals in the present paradigm. Specifically, it would be important to study combined effects of gaze behaviour on the one hand, and visual and/or vocal trustworthiness [45,46] on the other hand. An additional aspect for future studies is the important distinction between effects of gaze direction and of dynamic gaze shifts [6,9,47,48]. It seems likely that being stared at continually creates a very different impression from situations where a speaker turns her gaze toward you. Our stimuli did show a real gaze shift in the averted conditions, but this always occurred before speech onset. A more detailed investigation into the critical timing of gaze behaviour effects on listener trustworthiness remains an important challenge for future research.
